# Antithrombotic Therapy Duration after Patent Foramen Ovale Closure for Stroke Prevention: Impact on Long-Term Outcome

**DOI:** 10.1155/2022/6559447

**Published:** 2022-10-27

**Authors:** Joelle Kefer, Karlien Carbonez, Sophie Pierard, François-Pierre Mouthuy, Andre Peeters, Cedric Hermans, Catherine Lambert, Christophe DeMeester, Thierry Sluysmans, Agnes Pasquet

**Affiliations:** ^1^Division of Cardiology, Cliniques Universitaires Saint-Luc, Université Catholique de Louvain (UCLouvain), Brussels, Belgium; ^2^Pôle de Recherche Cardiovasculaire, Institut de Recherche Expérimentale et Clinique (IREC), Université Catholique de Louvain (UCLouvain), Brussels, Belgium; ^3^Division of Pediatric Cardiology, Cliniques Universitaires Saint-Luc, Université Catholique de Louvain (UCLouvain), Brussels, Belgium; ^4^Division of Neurology, Cliniques Universitaires Saint-Luc, Université Catholique de Louvain (UCLouvain), Brussels, Belgium; ^5^Division of Hematology, Haemostasis and Thrombosis Unit, Cliniques Universitaires Saint-Luc, Université Catholique de Louvain (UCLouvain), Brussels, Belgium

## Abstract

**Background:**

The optimal duration of antithrombotic therapy (ATT) after patent foramen ovale (PFO) closure remains under debate. This study sought to compare the clinical outcome of patients receiving antithrombotic agents for a short (6 months) versus extended (>6 months) period after the procedure.

**Methods:**

This was a retrospective cohort study using a propensity score matching analysis on 259 consecutive patients (131 males, 43 ± 10 years) undergoing PFO closure due to cryptogenic stroke, with complete follow-up (median duration of 10 [4–13] years). The outcome was compared between patients receiving short-term (Group short, *N* = 88) versus extended ATT (Group long, *N* = 171).

**Results:**

The PFO closure device was successfully implanted in all cases, with 3% of minor complications. After propensity score matching, there were no differences between Groups short and long in the rate of stroke (0.3 vs. 0.4% patient-year, *p*=1.00), bleeding (2 vs. 2% patient-year, *p*=0.17), and device thrombosis (0.3 vs. 0.1% patient-year; *p*=0.60). Univariate analysis showed that short-term ATT was not associated with an increased risk of recurrent stroke (HR: 1.271 [95% CI: 0.247–6.551], *p*=0.775) or prosthesis thrombus (HR: 0.50 [95% CI: 0.070–3.548], *p*=0.72). Kaplan–Meier analysis revealed similar overall survival in Group short and long (100 vs. 99 ± 1%, respectively; *p*=0.25).

**Conclusions:**

Short-term (6 months) ATT after PFO closure did not impair the clinical outcome, with a preserved low rate of recurrent stroke (0.3% patient-year) and device thrombosis (0.2% patient-year) at 10-year follow-up.

## 1. Introduction

**Figure 1:**
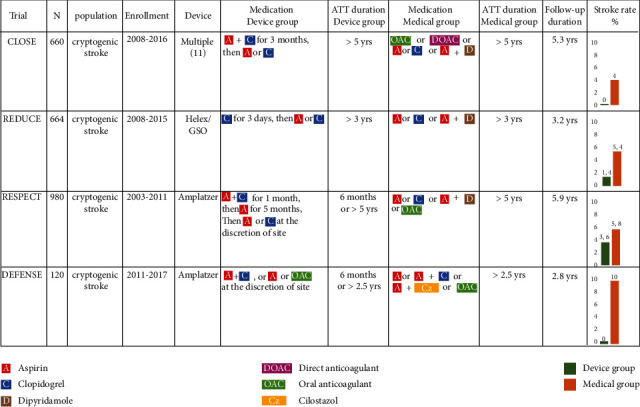
Recent randomized studies on PFO closure versus medical therapy after cryptogenic stroke: Rates of events and antithrombotic strategies. N = number of patients; ATT = antithrombotic therapy; GSO = Gore Cardioform Septal Occluder.

Recent randomized studies conducted with patients who had had cryptogenic stroke [1–4] have shown the positive impact of patent foramen ovale (PFO) closure vs. medical therapy to prevent recurrent ischemic stroke. In the periprocedural period, patients were mainly given antiplatelet therapy (single or dual) for a minimum of 3 months, which was continued for a highly variable duration of up to 5 years thereafter (Figure 1). After transcatheter atrial septal defect (ASD) closure, the European Society of Cardiology (ESC) guidelines [5] recommend an antiplatelet therapy for 6 months, which is the presumed time needed for endothelial coverage of the device [6]. Regarding PFO closure, a long-term antithrombotic therapy (ATT) is currently recommended, as for any patient in secondary prevention after an ischemic stroke [7, 8]. Yet, the optimal duration of ATT after PFO closure remains under debate.

Long-term medical therapy is supposed to improve thromboembolic protection, which could however be counterbalanced by an increased risk of adverse events and bleedings, as observed with extended dual antiplatelet therapy after coronary angioplasty [9] or with low-dose aspirin in primary prevention [10].

The objective of the present study was to compare the long-term outcome in a cohort of consecutive patients undergoing PFO closure after cryptogenic stroke, who received antithrombotic agents for a short (6 months) vs. extended (>6 months) period after the procedure.

## 2. Methods

### 2.1. Patients

**Figure 2:**
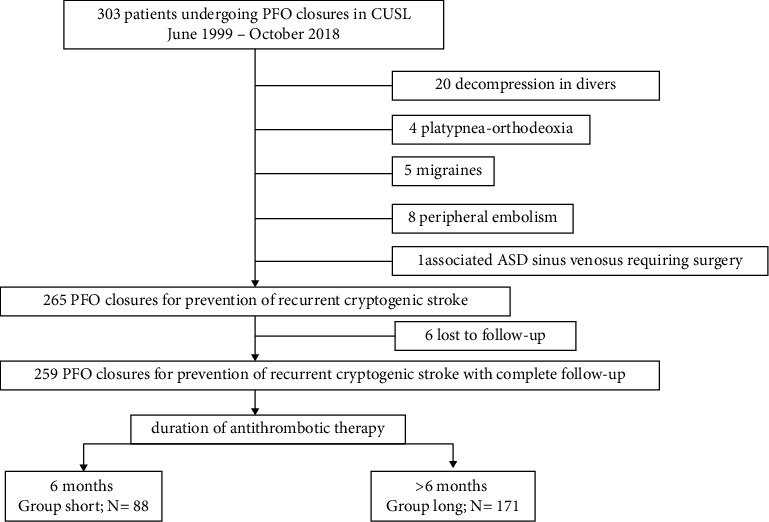
Study flow chart. ASD, atrial septal defect; CUSL, Cliniques universitaires Saint-Luc, PFO, patent foramen ovale.

Between June 1999 and October 2018, 303 consecutive patients underwent PFO closure at our institution. Among these, 265 underwent the procedure to prevent recurrent cryptogenic stroke (Figure 2) after an extensive workup including cerebral magnetic resonance imaging (MRI), angio-MRI of the circle of Willis, echo-Doppler of cervical arteries, at least 24-hour Holter monitoring, and transesophageal echocardiography (TEE), all of which were normal excepting for the ischemic lesion at MRI and PFO at TEE. The right-to-left shunt was identified by TEE and/or transcranial Doppler, with blood tests for thrombophilia screening performed (protein C or S deficiency, antithrombin deficiency, antiphospholipid antibodies, activated protein C resistance, factor V Leiden mutation, lupus anticoagulant, and prothrombin G20210A mutation). The patients were all assessed by a stroke-team, including a vascular neurologist, hematologist, echocardiographist, and invasive cardiologist, in order to precise the diagnosis of cryptogenic stroke and confirm the indication of transcatheter PFO closure.

### 2.2. Study Protocol

All PFO closure procedures were performed under general anesthesia and TEE guidance. Procedural success was considered in case of proper transfemoral delivery of the prosthesis in the interatrial septum without significant residual shunt, defined as no more than 2-mm jet width at TEE color Doppler at the intervention's end [11]. Periprocedural complications (from day 0 to hospital discharge) were defined according to the VARC-2 criteria [12]. Major complications included death, tamponade, myocardial infarction, disabling stroke, major bleeding (BARC 3 or 5) [13], major vascular damage, persistent arrhythmias (atrial fibrillation, conduction abnormalities), prosthesis embolization, and thrombosis. Minor complications included pericardial effusion not requiring surgery or drainage, minor bleeding (BARC 1 or 2), minor vascular damage, and nonpersistent (<24 hours) arrhythmias.

Before discharge, patients underwent clinical examination, 12-lead electrocardiogram, and transthoracic echocardiogram. Antithrombotic medications were prescribed at discharge for minimum 6 months. Patients with pre-existing thrombophilia received a preventive dosage of low-molecular-weight heparin associated with an antiplatelet agent for 1 month. A clinical and transthoracic echocardiographic follow-up was planned at 1, 6, and 12 months, and yearly thereafter. A TEE follow-up was recommended in case of recurrent stroke.

Late prosthesis dysfunction was defined as thrombus deposit, device dislocation/embolization, significant residual shunt, or pericardial effusion occurring during the follow-up period.

Clinical and procedural characteristics, periprocedural and long-term outcomes, as well as antithrombotic medications were prospectively collected into a dedicated database. Clinical events and ATT were determined from reviews of medical records or direct contact with the patient or referring physician. All neurological events were diagnosed by a neurologist and defined according to the relevant guidelines [14]. Informed consent was obtained from each patient, the study protocol was approved by our local ethics committee and conforms to the ethical guidelines of the 1975 declaration of Helsinki.

### 2.3. Statistical Analysis

Continuous variables were expressed as mean ± standard deviation when normally distributed, and as median and range when non-normally distributed. Categorical variables were presented as counts and percentages. Between-group comparisons were analyzed using the independent samples *t*-test for continuous variables and the chi-square or Fisher's exact test, where appropriate, for categorical variables.

Propensity score analysis and matching were built using the R software (version 4.0.3). Scores were computed using a 1 : 1 logistic regression model, with the response variable being the group of patients who stopped ATT at 6 months after PFO closure. The 5 covariables used to build the propensity score were age, hypertension, previous multiple stroke, factor V Leiden mutation, and antiphospholipid antibodies. The area under the receiver operating characteristic curve was 0.63 for the model built. The calculated propensity scores were then used to select pairs of patients with matched propensity scores in the 2 groups (1 : 1 match following the nearest neighbor matching rule) within a caliper of 0.15, using the matching package. Matched groups were compared using the paired *t*-test (absolute standardized differences test).

Estimates of freedom from death or adverse events were obtained using the Kaplan–Meier method. Univariate and multivariate analysis was carried out using the Cox proportional hazards method. A *p* value < 0.05 was considered statistically significant. Analyses were performed using the R software (version 4.0.3).

## 3. Results

### 3.1. Patients

The study included overall 259 consecutive patients (age 43 ± 10 years, 51% males) who underwent PFO closure after cryptogenic stroke and had a complete follow-up. Baseline characteristics are listed in Table 1.

Patients receiving ATT for a short period (6 months, Group short; *N* = 88) were younger and suffered less frequently from hypertension and prior multiple strokes than those undergoing extended ATT (>6 months, Group long; *N* = 171).

Thrombophilia was observed in 7% of the cohort patients, with no difference between Groups short and long (9% vs. 6%, *p*=0.47).

After matching, 86 pairs of matched patients were identified, resulting in similar baseline characteristics.

### 3.2. Procedural Characteristics

Procedural success was achieved in all cases (in one patient, a significant residual shunt due to an additional ASD required a repeated procedure with successful second prosthesis implantation, resulting in final complete closure). The median delay between cryptogenic stroke and intervention was 232 [137–420] days; atrial septal aneurysm was observed in 59% of cases; the type of prosthesis was mainly Amplatzer PFO Occluder (Abbott, Chicago, Illinois), with no relevant difference between both groups (Table 2). There were no death, tamponade, and major complication cases observed upon the periprocedural period. The rate of minor periprocedural complications was 3%, including two minor vascular events (one femoral fistula and one femoral pseudoaneurysm), four minor bleedings at the femoral puncture site, one nondisabling stroke, one transient ST segment modification due to air bubble embolization in the right coronary artery, and one transient atrial fibrillation that was treated by medications and resolved within 6 hours after the procedure. There was a significant reduction in the number of complications over time, from 7% among the first 100 patients to 1.2% thereafter (*p*=0.03).

Antithrombotic medications at discharge are being detailed in Table 2, with single antiplatelet therapy given to 65% of cases.

### 3.3. Follow-up

The mean duration of follow-up was 9 ± 5 years, and the median value was 10 years (interquartile range: 1281–4690 days), resulting in 2314 patient-years (Table 3).

During this follow-up period, four deaths occurred at a median time of 8 years after the procedure. The causes of death were cancer (*N* = 2), one suicide, and one ischemic stroke due to carotid occlusion occurring 4.4 years after PFO closure. Six additional patients experienced a recurrent ischemic stroke at a mean delay of 3.1 ± 1.5 years after the procedure. This means that the recurrent stroke rate was estimated at 0.3% patient-year. One was related to an atrial fibrillation, one was due to PFO-device thrombosis, whereas for four strokes, no cause could be identified. Therefore, the rate of recurrent cryptogenic stroke was estimated at 0.2% patient-year. A TEE investigation was performed in all these patients, which revealed a good position and function of the PFO device excepting for residual shunt detected in one case, and a device thrombosis occurring during cardiac arrest on account of anaphylactic shock at 9 months postprocedure. Five of these recurrent strokes (71%) were considered nondisabling. Details of recurrent strokes are provided in Table 4.

No major bleedings were observed during the follow-up period, while 48 minor bleedings occurred, yet without any significant difference between both groups (14 vs. 22% in Groups short and long, respectively, *p*=0.17). The bleeding causes were subcutaneous hematoma (*N* = 31), epistaxis (*N* = 10), dental (*N* = 4), hemarthrosis (*N* = 1), gynecologic (*N* = 1), and hemorrhoidal (*N* = 1) in nature.

ATT at last follow-up in Group long mainly comprised aspirin (90%), followed by anticoagulants (7%), dual antiplatelet therapy (2%), and clopidogrel (1%).

The duration of ATT was significantly shorter in Group short than in Group long (184 ± 27 vs. 2628 ± 1993 days, *p* < 0.001). Univariate analysis revealed that ATT discontinuation at 6 months following PFO closure was not associated with an increased risk of recurrent stroke (hazard ratio [HR]: 1.271 [95% CI: 0.247–6.551], *p*=0.775). The only characteristic associated with an increased risk of recurrent stroke was thrombophilia, which was exclusively significant for the factor V Leiden mutation (HR: 15.64 [95% CI: 1.9-128-59], *p*=0.01) and antiphospholipid antibody syndrome (HR: 39.76 [95% CI: 4.37–361.52], *p*=0.001). Nevertheless, among these thrombophilia patients, a short-duration ATT did not increase the risk of recurrent stroke during follow-up (HR: 0.68 [95% CI: 0.043–10.977], *p*=0.79).

**Figure 3:**
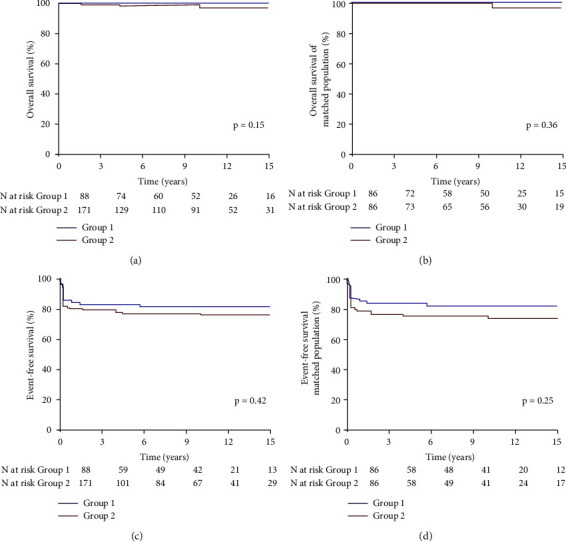
Kaplan–meier analysis showing the comparison between groups of the overall and event-free survival in the total cohort and in the matched population. Group 1, Group short; Group 2, Group long; ATT, antithrombotic therapy.

Figure 3 illustrates the Kaplan–Meier analysis. At 10-year follow-up, overall survival and event-free survival were 99 ± 1% and 99 ± 2% in the total cohort and 100% and 99 ± 2% in the matched population, respectively, with no significant difference between Groups short and long (100 vs. 99 ± 1% and 98 ± 4 vs. 99 ± 3%, respectively, *p*=0.25).

At systematic echocardiographic follow-up, four (two in each group) thrombotic deposits on the PFO prosthesis (three Cardia Star and one Occlutech PFO devices) were detected at a median time of 349 (interquartile range: 226–867) days after the procedure. Of these four patients, two were totally asymptomatic, whereas the two others suffered from a recurrent neurologic event, including one transient ischemic attack and one stroke. The thrombus resolved under anticoagulant therapy among three of them, whereas it was still persistent 6 months after treatment in one of these patients, eventually requiring surgery for extracting the thrombus and partially mal-apposed Cardia Star prosthesis. Three of these patients did not undergo any ATT at the time of thrombus discovery, while two of them displayed thrombophilia, including one protein S deficiency and one antiphospholipid antibody. Details of thrombotic deposits on the PFO prosthesis are provided in Table 5. Univariate analysis revealed that thrombophilia was associated with an increased risk of device thrombosis (HR: 16.96 [95% CI: 2.374–121.241], *p*=0.005), whereas a short-duration ATT was not associated with the risk of device thrombosis either in the total population (HR: 0.50 [95% CI: 0.070–3.548], *p*=0.48) or among thrombophilia patients (HR: 0.60 [95% CI: 0.038–9.675], *p*=0.72). On multivariate analysis (Table 6), thrombophilia was an independent predictor of both, recurrent stroke (HR: 6.35 [95% CI: 1.22–32.98], *p*=0.028) and device thrombosis (HR: 29.07 [95% CI: 3.28–257.6], *p*=0.002).

During the follow-up period, neither delayed device dislocation/embolization nor pericardial effusion occurred. Overall, the rate of late prosthesis dysfunction was estimated at 0.2% patient-year, without any difference noted between Groups short and long (0.3% and 0.1% patient-year, respectively, *p*=0.60).

## 4. Discussion

**Figure 4:**
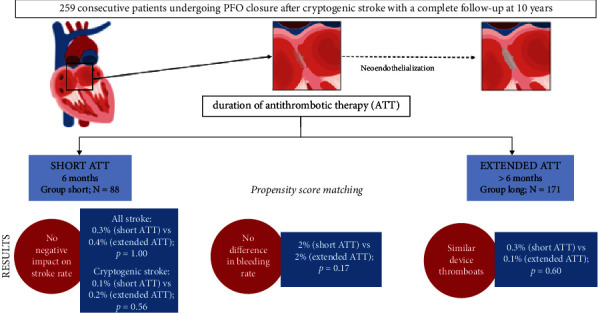
Central illustration. PFO closure to prevent recurrent cryptogenic stroke: Overview of the study methodology and main results. PFO, patent foramen ovale; ATT, antithrombotic therapy.

The main findings of the current study (Figure 4. Central illustration) are, as follows:

1. In strongly selected patients undergoing transcatheter PFO closure due to cryptogenic stroke, the procedure is deemed safe and effective in stroke prevention (0.3% patient-year), and this up to 10 years of follow-up.
2. Discontinuation of ATT at 6 months after the procedure did not impair the clinical outcome, nor did it increase the risk of recurrent stroke.
3. Late prosthesis dysfunction is rare (0.2% patient-year), and it is not impacted by ATT cessation at 6 months after the procedure.

### 4.1. Safety and Efficiency of Transcatheter PFO Closure in Stroke Prevention at Long Term

The first randomized studies including patients affected by transient ischemic attack (TIA) and stroke [15, 16] failed to demonstrate a significant reduction in recurrent stroke after PFO closure, in comparison with medical therapy, while reporting a high rate of major procedural complications (>3%) and overall complications estimated at around 5%. In our study, there were only 3% of minor complications, and this rate was significantly reduced after the first 100 patients (from 7% to 1.2%). This low rate of procedural complications is in line with more recent studies [1–4], clearly reflecting the operators' learning curve. Randomized studies including only patients with cryptogenic stroke and excluding those with TIA [1–4] have demonstrated the superiority of PFO closure over medical therapy, with a variable impact on stroke rates. This is most likely due to the different types of devices used and their effective shunt closure capacities, but also to the follow-up duration, ranging from 2.8 to 5.9 years. The relevance of extended follow-up was highlighted by the RESPECT trial's long-term analysis, [3] demonstrating a significant benefit in favor of PFO closure, whereas the first publications [17] failed to demonstrate such a difference. The latter is most probably related to the shorter patient follow-up (median value: 2.6 versus 5.9 years, respectively). Wintzer-Wehekind [18] reported a stroke rate estimated at 0.08% patient-year at 12-year follow-up among 201 unmatched patients, with a tendency of thrombophilia to effectively increase ischemic events. Likewise, our study revealed similarly low stroke (0.3% patient-year) and low cryptogenic stroke (0.2% patient-year) rates at 10 years, using propensity score matching of a cohort of 259 consecutive patients. Thrombophilia was observed in 7% of the population, which was significantly associated with an increased risk of recurrent stroke. These observations are similar to those reported by Liu et al. [19] that revealed thombophilia to increase the risk of recurrent events (HR: 1.85), and PFO closure to be superior to medical therapy in terms of stroke prevention (HR: 0.25) in such patients. A meta-analysis including eleven studies [20] confirmed that thrombophilia was associated with an increased recurrence risk in PFO patients with cryptogenic stroke (OR: 2.41), and that this risk lost statistical significance after PFO closure (odds ratio (OR): 2.07, [95% CI: 0.95–4.48]). The authors stated that the studies' heterogeneity precluded strong conclusions to be drawn with respect to ATT duration following PFO closure.

### 4.2. Impact of ATT Discontinuation

ATT duration in randomized studies [1–4] ranged from 6 months to >5 years, and long-term ATT after PFO closure is currently recommended, even though the impact of discontinuing ATT after 6 months is not yet clear. In a histological analysis of explanted devices from human beings, Foth et al. [6] demonstrated that neo-endothelialization was observed in all specimens following implantation times of 10 weeks or more. Like in transcatheter ASD closure, for which ATT and endocarditis prophylaxis are given for 6 months, which is the presumed time needed for a complete endothelial coverage of the device, ATT could be stopped 6 months after PFO closure. In our study, patients underwent a strong selection process performed by a dedicated stroke-team in order to confirm the cryptogenic stroke. Therefore, our population was at very low cardiovascular risk, as they were younger and less frequently diabetics as respect to other series [2–4, 18]. After propensity score matching, the stroke rate was similarly low among those who stopped than among those who continued ATT at 6 months (0.3 and 0.4% patient-year, respectively, *p*=1.00) at 10 years of follow-up. Wintzer-Wehekind et al. [21] published a series of 46 matched patients who stopped ATT within one year of PFO closure, revealing no difference in ischemic events at 7 years of follow-up, in comparison with 120 patients on prolonged ATT. Nevertheless, these authors showed a significant increase in major bleedings in the event of prolonged ATT. De-escalation of antiplatelet therapy after coronary angioplasty is currently under investigation, on account of the increased bleeding and mortality risk encountered under prolonged dual antiplatelet therapy [9], especially in high-bleeding-risk patients. These latter patients can be safely treated with one-month dual antiplatelet therapy following new-generation drug-eluting stent implantation [22]. Given in primary prevention to moderate-risk patients [10], low-dose aspirin prescribed as monotherapy was revealed to significantly increase the risk of bleeding, without any protective effect in terms of ischemic events. In our population of very-low-risk patients, it can be assumed that a successful PFO closure returns them to a primary prevention stage, without any additional benefit gained by pursuing long-term aspirin administration. It must be noted here that our observations clearly showed that ATT discontinuation at 6 months postintervention did not increase the risk of recurrent strokes, nor did it impair the clinical outcome at up to 10 years of follow-up after PFO closure. Given that the procedure is usually performed among young patients, a lifelong ATT could potentially expose them to bleeding events and medications' undesirable effects when given during such a long treatment period. These findings suggest that in strongly selected patients undergoing PFO closure due to cryptogenic strokes, ATT cessation at 6 months after a successful procedure would be a quite reasonable strategy. An extended ATT duration might be considered in patients at higher CV risk profile undergoing PFO closure, where the “cryptogenic” status is less obvious, but still undergoing the procedure in real-life settings.

### 4.3. Late Prosthesis Dysfunction

The rate of prosthesis dysfunction was reported to be as high as 5% by Hornung et al. [23]. In their study, these authors randomized patients according to three different devices and showed significant differences in terms of thrombus and residual shunts, which were all in favor of the Amplatzer device, as compared with the Helex, CardioSEAL, and STARflex systems. Rigatelli et al. [24] reported a PFO-device thrombosis rate at 0.2% at 10 years of follow-up, while including 453 patients that were mainly treated with an Amplatzer device (75% cases) and ceased ATT after 6 months.

In the present study, late prosthesis dysfunction was similarly low (0.2% patient-year), including one significant residual shunt and four thromboses, which were more commonly observed among thrombophilia patients (11% vs. 0.8% in the absence of thrombophilia, *p*=0.02), yet without being impacted by ATT duration. All thrombus deposits were detected on non-Amplatzer devices. These observations suggest to be careful with thrombophilic patients undergoing PFO closure, and to treat them with low-thrombogenic devices.

### 4.4. Study Limitations

This is a retrospective analysis of a single center registry, with inherent limitations. This is a nonrandomized study on a small sample size with a low event rate, so underpowered, and bias still remains after propensity score matching.

At follow-up, the evaluation of residual shunting and device-thrombus was performed using transthoracic echocardiography, which may result in an underestimation of their actual incidences. Nevertheless, a TEE was performed in each patient that experienced a recurrent stroke, with only one significant residual shunt and one thrombus detected among these cases. Even if transthoracic echocardiographic follow-up is less complete and has a lower sensitivity than TEE, it is less invasive, more available, and properly reflects the way in which most patients are followed-up after PFO closure in real-life settings.

## 5. Conclusions

The current study has shown that among strongly selected patients at low cardiovascular risk, the rate of recurrent stroke and cryptogenic stroke after successful transcatheter PFO closure is very low (0.3 and 0.2% patient-year, respectively) at a 10-year follow-up. ATT discontinuation at 6 months versus extended ATT did not increase the risk of recurrent strokes, device thrombosis, nor did it impair the patient outcome.

## Figures and Tables

**Table 1:** Baseline characteristics of patients.

Characteristic		Before PS matching				After PS matching		
		All	Gr short	Gr long	*p*	Gr short	Gr long	*p*
		*N* = 259	*N* = 88	*N* = 171	value	*N* = 86	*N* = 86	value
Age (yrs)	mean ± SD	43 ± 10	40 ± 10	44 ± 10	0.0001	40 ± 10	41 ± 10	0.51
Gender	Male	131 (51)	49 (56)	82 (48)	0.24	48 (56)	37 (43)	0.09
Smoking	*n* (%)	105 (40)	33 (38)	72 (42)	0.48	32 (37)	38 (44)	0.35
Hypertension	*n* (%)	48 (19)	10 (11)	38 (22)	0.03	10 (12)	10 (13)	1.0
Diabetes	*n* (%)	6 (2)	1 (1)	5 (3)	0.37	1 (1)	3 (3)	0.31
LDL-cholesterol (mg/dl)	mean ± SD	123 ± 37	121 ± 36	124 ± 38	0.57	120 ± 36	122 ± 40	0.81
Carotid artery disease	*n* (%)	0	0	0	—	0	0	—
Prior atrial fibrillation	*n* (%)	1 (0.4)	0	1 (1)	0.47	0	0	—
Left ventricular ejection fraction <50%	*n* (%)	0	0	0	—	0	0	—
Pulmonary hypertension	*n* (%)	0	0	0	—	0	0	—
Migraine	*n* (%)	103 (40)	35 (40)	68 (40)	1.00	35 (41)	37 (43)	0.76
Previous transient ischemic attack	*n* (%)	29 (11)	9 (10)	20 (12)	0.72	8 (9)	8 (9)	1.0
Prior multiple strokes	*n* (%)	23 (9)	3 (3)	20 (12)	0.03	3 (3)	0	0.08
Thrombophilia	*n* (%)	19 (7)	8 (9)	11 (6)	0.47	9 (10)	4 (5)	0.15
Protein C deficiency	*n* (%)	2 (1)	2 (2)	0	0.05	2 (2)	0	0.16
Protein S deficiency	*n* (%)	7 (3)	3 (3)	4 (2)	0.62	3 (3)	1 (1)	0.31
Factor V Leiden	*n* (%)	4 (2)	1 (1)	3 (2)	0.7	1 (1)	1 (1)	1.0
Lupic Antibodies	*n* (%)	1 (0.4)	1 (1)	0	0.16	1 (1)	0	0.32
Antiphospholipid Antibodies	*n* (%)	2 (1)	1 (1)	1	0.63	1 (1)	0	0.32
Prothrombin mutation	*n* (%)	5 (2)	1 (1)	4 (2)	0.51	1 (1)	1 (1)	1.0
Antithrombin III	*n* (%)	0	0	0	—	0	0	—
History of deep vein thrombosis	*n* (%)	16 (6)	5 (6)	11 (6)	0.81	5 (6)	6 (7)	0.76
Hormone therapy	*n* (%)	79 (31)	24 (27)	55 (32)	0.42	23 (27)	32 (37)	0.14

Gr = Group; LDL = low-density lipoprotein; PS = propensity score; SD = standard deviation; yrs = years.

**Table 2:** Periprocedural outcome.

Characteristic		Before PS matching				After PS matching		
		All	Gr short	Gr long	*p*	Gr short	Gr long	*p*
		*N* = 259	*N* = 88	*N* = 171	value	*N* = 86	*N* = 86	value
Delay between stroke and cath (days)	median [IQR]	232 [137–420]	214 [127–360]	241 [150–440]	0.46	214 [127–365]	243 [153–434]	0.49
Atrial septal aneurysm	*n* (%)	152 (59)	47 (53)	105 (61)	0.23	46 (53)	52 (60)	0.44
Prosthesis type; size (mm)								
Amplatzer; 25 [25–30]	*n* (%)	105 (41)	32 (36)	73 (43)	0.35	32 (37)	29 (34)	0.75
Cardia Star; 30 [25–30]	*n* (%)	99 (38)	37 (42)	62 (36)	0.42	36 (42)	35 (41)	1.00
Starflex; 28 [28–33]	*n* (%)	11 (4)	6 (7)	5 (3)	0.19	6 (7)	2 (2)	0.28
Gore; 28 [25–30]	*n* (%)	6 (2)	1 (1)	5 (3)	0.67	1 (1)	3 (3)	0.62
Premere; 25 [25–25]	*n* (%)	13 (5)	2 (2)	11 (6)	0.23	2 (2)	7 (8)	0.17
Occlutech*;* 25 [25–30]	*n* (%)	23 (9)	9 (10)	14 (8)	0.65	8 (9)	9 (10)	1.00
PFM Nit Occlud; 22 [22–25]	*n* (%)	2 (1)	1 (1)	1 (1)	1.00	1 (1)	1 (1)	1.00
Prosthesis size (mm)	media*n* [IQR]	25 [25–30]	25 [25–30]	25 [25–30]	0.21	25 [25–30]	25 [25–30]	0.35
Complication	*n* (%)	9 (3)	2 (2)	7 (4)	0.72	2 (2)	5 (6)	0.44
Minor vascular	*n* (%)	2 (1)	0	2 (1)	0.55	0	0	—
Minor bleeding	*n* (%)	4 (2)	1 (1)	3 (2)	1.00	1 (1)	3 (3)	0.62
Non disabling stroke	*n* (%)	1 (0.3)	0	1 (1)	1.00	0	1 (1)	1.00
Transient ischemic attack	*n* (%)	0	0	0	—	0	0	—
Transient ST segment modification	*n* (%)	1 (0.3)	0	1 (1)	1.00	0	1 (1)	1.00
Death	*n* (%)	0	0	0	—	0	0	—
Transient atrial fibrillation	*n* (%)	1 (0.3)	1 (1)	0	1.00	1 (1)	0	1.00
Antithrombotic treatment at discharge								
Aspirin	*n* (%)	164 (63)	54 (61)	110 (64)	0.68	54 (63)	52 (60)	0.87
Clopidogrel	*n* (%)	5 (2)	1 (1)	4 (2)	0.66	1 (1)	2 (2)	1.00
Dual antiplatelet therapy	*n* (%)	87 (33.5)	32 (36)	55 (32)	0.58	30 (35)	30 (35)	1.00
Anticoagulants	*n* (%)	1 (0.5)	1 (1)	0	0.34	1 (1)	0	1.00
Aspirin *+* anticoagulants	*n* (%)	2 (1)	0	2 (1)	0.55	0	2 (2)	0.50

Gr = Group; IQR = interquartile range; PS = propensity score.

**Table 3:** Follow-up after PFO closure.

Characteristic		Before PS matching				After PS matching		
		All	Gr short	Gr long	*p*	Gr short	Gr long	*p*
		*N* = 259	*N* = 88	*N* = 171	value	*N* = 86	*N* = 86	value
Duration of antithrombotic therapy (days)	median [IQR]	587 [194–2856]	184 [181–194]	1519 [594–3987]	<0.001	184 [181–194]	2390 [702–4172]	<0.001
Duration of aspirin	median [IQR]	630 [194–2315]	184 [181–194]	1324 [623–3007]		184 [181–194]	2125 [692–3307]	
Duration of clopidogrel	median [IQR]	434 [222–837]	184	635 [381–2372]		184	529 [376–683]	
Duration of dual antiplatelet therapy	median [IQR]	370 [194–4330]	184 [180–197]	4036 [496–5322]		184 [180–199]	4238 [858–5399]	
Duration of anticoagulants	median [IQR]	185	185	—		185	—	
Duration of aspirin + anticoagulants	median [IQR]	3711 [2221–5201]	—	3711 [2221–5201]		—	3711 [2221–5201]	
Duration of follow-up (years)	median [IQR]	10 [4–13]	10 [4–14]	9 [3–13]	0.52	10 [4–14]	11 [6–14]	0.16
Major adverse events								
Death	*n* (%)	4 (1.5)	0 (0)	4 (2.3)	0.15	0 (0)	1 (1)	0.32
Cardiovascular death	*n* (%)	1 (0.4)	0 (0)	1 (0.6)	1.00	0 (0)	0 (0)	—
Stroke	*n* (%)	7 (3)	2 (3)	5 (3)	0.83	2 (2)	3 (3)	1.00
Ischemic stroke	*n* (%)	8 (3)	3 (3)	5 (3)	0.83	3 (3)	3 (3)	1.00
Hemorrhagic stroke	*n* (%)	0 (0)	0 (0)	0 (0)	—	0 (0)	0 (0)	—
Cryptogenic stroke	*n* (%)	4 (2)	1 (1)	3 (2)	0.70	1 (1)	2 (2)	0.56
Major bleeding	*n* (%)	0 (0)	0 (0)	0 (0)	—	0 (0)	0 (0)	—
Other adverse events								
Minor bleeding	*n* (%)	48 (19)	13 (15)	35 (20)	0.27	12 (14)	19 (22)	0.17
BARC-1	*n* (%)	44 (17)	12 (14)	32 (19)	0.38	11 (13)	16 (19)	0.40
BARC-2	*n* (%)	4 (2)	1 (1)	3 (2)	1.00	1 (1)	3 (3)	0.62
Transient ischemic attack	*n* (%)	9 (3)	3 (3)	6 (4)	1.00	3 (3)	3 (3)	1.00
Atrial fibrillation (paroxysmal)	*n* (%)	17 (7)	3 (3)	14 (8)	0.18	3 (3)	7 (8)	0.33

Gr = Group; IQR = interquartile range; PS = propensity score.

**Table 4:** Details of recurrent strokes.

Age at recurrent stroke	Delay	Type of stroke	Cause identified	Prosthesis type	ATT at time of event	TEE shunt/thrombus	Atrial fibrillation	Outcome
56 years	4 years	Ischemic	No	Cardia	Aspirin	No/No	No	Recovery
43 years	1.1 year	Ischemic	No	Cardia	Aspirin	Yes/No	No	Recovery
52 years	4.4 years	Ischemic	Carotid occlusion	Cardia	Anticoagulant	Not evaluated	No	Death
58 years	1.4 year	Ischemic	No	Amplatzer	None	No/No	No	Recovery
30 years	0.8 year	Ischemic	PFO device thrombosis	Occlutech	None	No/Yes	No	Persistant illness
43 years	0.5 year	Ischemic	Atrial fibrillation	Occlutech	Aspirin	No/No	Yes	Recovery
21 years	0.2 year	Ischemic	No	Cardia	Aspirin	No/No	No	Recovery

ATT = antithrombotic therapy; PFO = patent foramen ovale; TEE = transesophageal echocardiography.

**Table 5:** Details of thrombotic deposits on the PFO prosthesis.

Patient	Prosthesis type	Delay (days)	ATT at discharge	ATT at time of event	Thrombophilia	Symptoms	Medical Treatment	Thrombus outcome	Patient outcome
1	Cardia Star 25mm	405	DAPT	None	No	No	Anticoagulation	Resolved	Good
2	Cardia Star 30mm	2252	Aspirin	None	No	TIA	Anticoagulation	Surgical removal	Good
3	Occlutech 35mm	293	Aspirin	None	Yes (Ac APL)	Stroke	Anticoagulation	Resolved	Persistant illness
4	Cardia Star 35mm	26	Aspirin	Aspirin	Yes (Prot S)	No	Anticoagulation	Resolved	Good

PFO = patent foramen ovale; Delay = time between procedure of PFO closure and event; ATT = antithrombotic therapy; DAPT = dual antiplatelet therapy; Ac APL = antiphospholipid antibodies; Prot S = protein S deficiency; TIA = transient ischemic attack.

**Table 6:** Univariate and multivariate analysis for predictors of stroke and device thrombosis according to Cox models.

Event Parameter	Stroke		Device thrombosis	
	HR [95% CI]	*p* value	HR [95% CI]	*p* value
*UNIVARIATE COX ANALYSIS*				
Age (years)	1.00 [0.93–1.07]	0.93	0.94 [0.85–1.03]	0.18
Gender (male)	0.95 [0.24–3.78]	0.94	2.94 [0.31–28.24]	0.35
Smoking	4.32 [0.87–21.43]	0.07	4.31 [0.45–41.47]	0.21
Hypertension	2.70 [0.65–11.32]	0.17	1.48 [0.15–14.21]	0.73
Migraine	0.44 [0.09–2.20]	0.32	0.45 [0.05–4.36]	0.49
Previous TIA	1.10 [0.14–8.95]	0.93	—	—
Previous multiple Stroke	1.46 [0.18–11.88]	0.72	10.44 [1.47–74.1]	0.02
History of deep vein thrombosis	2.17 [0.27–17.64]	0.47	—	—
Hormone therapy	0.74 [0.15–3.68]	0.71	0.73 [0.08–7.06]	0.79
Thrombophilia	4.03 [0.81–19.97]	0.09	16.96 [2.23–121.24]	0.005
Protein C deficiency	—	—	—	—
Protein S deficiency	—	—	16.8 [1.72–164.18]	0.02
Factor V Leiden	15.64 [1.9–128.59]	0.01	—	—
Antiphospholipid Antibodies	39.76 [4.37–361.52]	0.001	77.94 [6.89–881.15]	<0.001
Antithrombin III	—	—	—	—
Lupic Antibodies	—	—	—	—
Short ATT	1.271 [0.24–6.55]	0.77	0.50 [0.07–3.54]	0.72
Amplatzer device	0.290 [0.03–2.44]	0.25	2.48 [0.01–5.56]	0.99

*MULTIVARIATE COX ANALYSIS*				
Smoking	3.75 [0.73–19.34]	0.12	—	—
Previous multiple Stroke	—	—	19.57 [2.25–170.0]	0.007
Thrombophilia	6.35 [1.22–32.98]	0.03	29.07 [3.28–257.6]	0.002

## Data Availability

The data that support the findings for this study are available from the corresponding author upon request.

## Abbreviations

ASD: Atrial septal defect
ATT: Antithrombotic therapy
HR: Hazard ratio
MRI: Magnetic resonance imaging
PFO: Patent foramen ovale
TEE: Transesophageal echocardiography.
